# Detection of *Klebsiella pneumonia* DNA and ESBL positive strains by PCR-based CRISPR-LbCas12a system

**DOI:** 10.3389/fmicb.2023.1128261

**Published:** 2023-02-09

**Authors:** Shang Wang, Shan Wang, Ying Tang, Guoyu Peng, Tongyu Hao, Xincheng Wu, Jiehong Wei, Xinying Qiu, Dewang Zhou, Shimao Zhu, Yuqing Li, Song Wu

**Affiliations:** ^1^Institute of Urology, The Third Affiliated Hospital of Shenzhen University, Shenzhen, China; ^2^Shenzhen Institute of Synthetic Biology, Shenzhen Institutes of Advanced Technology, Chinese Academy of Sciences, Shenzhen, China; ^3^Teaching Center of Shenzhen Luohu Hospital, Shantou University Medical College, Shantou, China; ^4^Medical Laboratory of Shenzhen Luohu People’s Hospital, Shenzhen, China; ^5^School of Medicine, Anhui University of Science and Technology, Huainan, China; ^6^Kobilka Institute of Innovative Drug Discovery, School of Medicine, The Chinese University of Hong Kong, Shenzhen, Guangdong, China; ^7^South China Hospital, Health Science Center, Shenzhen University, Shenzhen, China

**Keywords:** CRISPR-Cas, nucleic acid detection, *Klebsiella pneumonia* (*K. pneumonia*), Extended-spectrum β-lactamases (ESBL), SHV

## Abstract

**Introduction:**

*Klebsiella pneumonia* (*K. pneumonia*) is a Gram-negative bacterium that opportunistically causes nosocomial infections in the lung, bloodstream, and urinary tract. Extended-spectrum β-Lactamases (ESBLs)-expressed *K. pneumonia* strains are widely reported to cause antibiotic resistance and therapy failure. Therefore, early identification of K. pneumonia, especially ESBL-positive strains, is essential in preventing severe infections. However, clinical detection of *K. pneumonia* requires a time-consuming process in agar disk diffusion. Nucleic acid detection, like qPCR, is precise but requires expensive equipment. Recent research reveals that collateral cleavage activity of CRISPR-LbCas12a has been applied in nucleic acid detection, and the unique testing model can accommodate various testing models.

**Methods:**

This study established a system that combined PCR with CRISPR-LbCas12a targeting the *K. pneumoniae* system. Additionally, this study summarized the antibiotic-resistant information of the past five years’ *K. pneumoniae* clinic cases in Luohu Hospital and found that the ESBL-positive strains were growing. This study then designs a crRNA that targets *SHV* to detect ESBL-resistant *K. pneumoniae*. This work is to detect *K. pneumoniae* and ESBL-positive strains’ nucleic acid using CRISPR-Cas12 technology. We compared PCR-LbCas12 workflow with PCR and qPCR techniques.

**Results and Discussion:**

This system showed excellent detection specificity and sensitivity in both bench work and clinical samples. Due to its advantages, its application can meet different detection requirements in health centers where qPCR is not accessible. The antibiotic-resistant information is valuable for further research.

## Introduction

*Klebsiella pneumonia* (*K. pneumonia*) is a family of Gram-negative bacteria that causes nosocomial infections in the bloodstream, wound, and urinary tract (Magill et al., 2014). Hypervirulent *K. pneumoniae* strains, such as K1, K2, and K5, have emerged worldwide and caused severe infections, including liver abscess and pneumonia, with a mortality rate as high as 20–30% (Podschun and Ullmann, 1998).

β-Lactamases (ESBLs) can degrade β-Lactam antibiotics into non-effective compounds, thus resulting in drug-resistant strains (Bush and Bradford, 2016). However, due to the excessive use of β-Lactam antibiotics, the prevalence of ESBL-producing *K. pneumonia* has primarily increased. To date, ESBL-producing *K. pneumonia* contributes to nearly 45% of *K. pneumoniae* nosocomial infections (Miftode et al., 2021) and 43% in the intensive care unit (Paterson et al., 2004; Calbo et al., 2011). More strikingly, ESBL-positive strains result in significantly higher mortality (Miftode et al., 2021); a recent study reported that ESBL-producing *K. pneumonia* is associated with over 55% mortality (Starzyk-Luszcz et al., 2017). Despite various genes encoding ESBLs, most ESBLs were derived from one or two amino acid substitutions of SHV-1 and TEM-1 (Ramdani-Bouguessa et al., 2011; Ben Achour et al., 2014). *SHV*-type ESBLs almost evolved from *SHV-1*; for example, *SHV-2* harbors G238S (Zhong et al., 2021). Over 100 *SHV* variants have been found,^1^ and most of which are associated with ESBL-positive strains. Until 2016, the *SHV*-type ESBLs only accounted for 10% ESBLs. However, the majority are found in *K. pneumoniae* (Castanheira et al., 2016). Thus, the detection of *SHV* is valuable in identifying ESBLs in *K. pneumoniae*.

*Klebsiella pneumonia* colonies derived from clinical samples under 24–48 h of 37°C incubation culture after disk diffusion present features that could be diagnosed by well-trained personnel (Wagner et al., 2016). Usually, 1 week is required to determine the specific antibiotic resistance of bacterial strain (Giske et al., 2022). Despite the low cost and the simplicity of operation, disk diffusion is time-consuming and sometimes produces false-negative results especially for atypical colonies (Johnson et al., 2006; Hutchison et al., 2018). In contrast, nucleic acid detection methods that detect *K. pneumoniae*-specific DNA fragments are more advantageous. For example, quantitative real-time PCR or qPCR is the most widely used one (Hyun et al., 2019). The *K. pneumonia* and specific drug-resistant strains can be determined by qPCR (Yan et al., 2021). For ESBL testing, *SHV-1*, *TEM*, and *CTX-M* gene DNA fragments are used (Souverein et al., 2017). However, qPCR requires expensive equipment, which restricts its application in the healthcare center.

The CRISPR-Cas systems have been discovered to cleave target DNA or RNA under the guidance of crRNA in a base-pairing manner (Yan et al., 2019). Recent research shows that Cas13 and Cas12 exhibit collateral cleavage activity that could degrade probes if crRNA perfectly base-pairs targeted RNAs or DNAs (Chen et al., 2018; Gootenberg et al., 2018). Combined with DNA amplification and signal detection methods, Cas12 and Cas13 have been applied to detect nucleic acid (Li et al., 2019). The detection workflow only requires 37°C incubation and does not rely on complicated equipment. To combat COVID-19, lots of nucleic acid detection workflow based on Cas13 and Cas12 have been developed (Kostyusheva et al., 2021; Nouri et al., 2021). Both Cas12 and Cas13 require no expensive equipment and are compatible with various amplification or detection methods.

**Figure 1:**
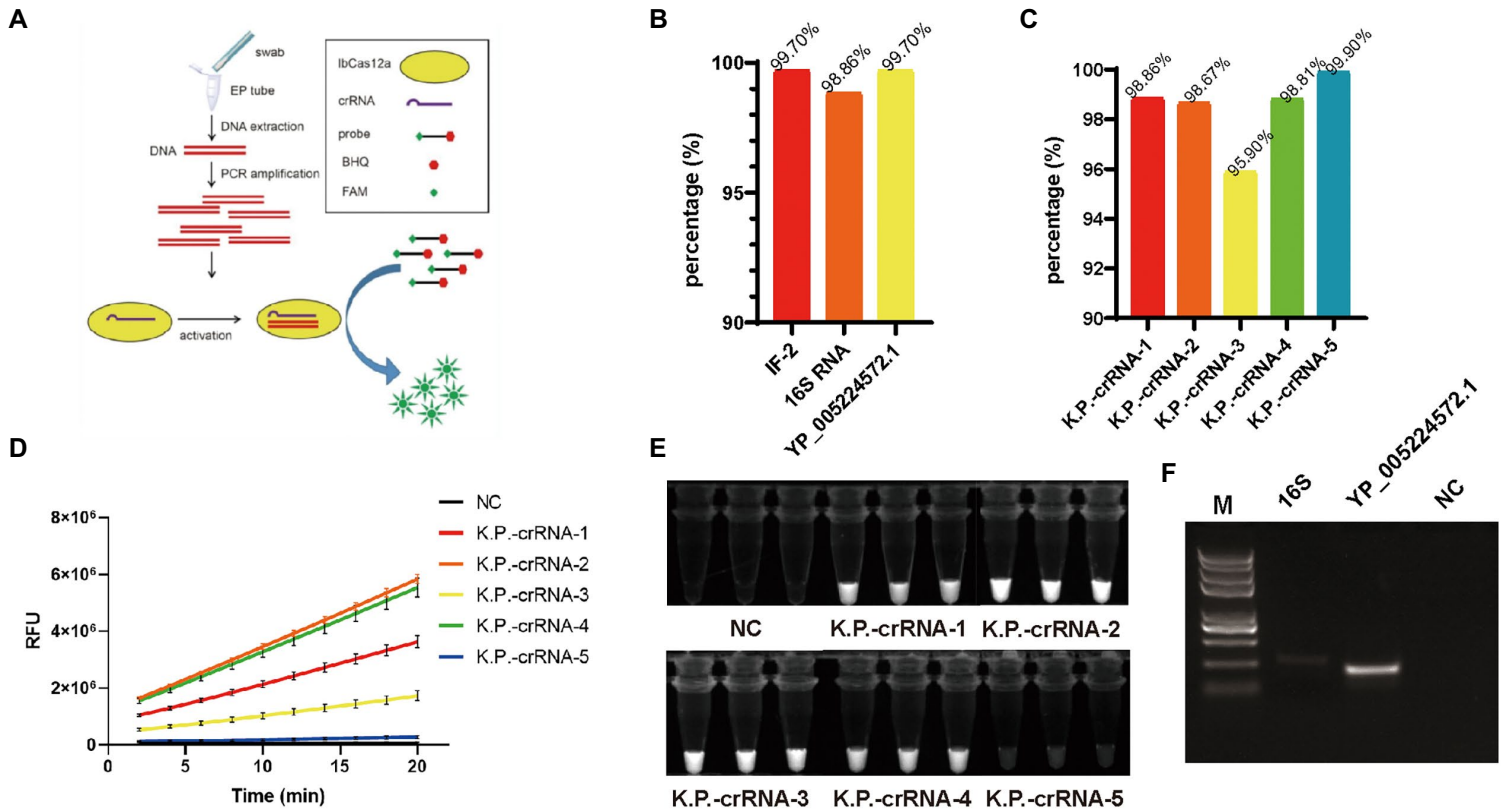
**(A)** Graphical illustration of PCR-LbCas12a detecting *Klebsiella pneumonia* nucleic acid. (**B)** The proportion of *IF-216SRNA* and *YP_005224572.1* in the 2024 *K. pneumonia* genomes. **(C)** The percentage of each K.P.-crRNA hitting the 2024 *K. pneumonia* genomes. **(D)** FAM signal of five K.P.-crRNA activity in LbCas12a reaction. **(E)** PCR-LbCas12a reaction products of **(D)** were photographed under UV activation. **(F)** The PCR amplification efficiency of primers targeting*16S* and *YP_005224572.1*.

Despite these advantages, the CRISPR-Cas12 detection method targeting *K. pneumonia* has not been reported. We established a sensitive nucleic acid detection method based on CRISPR-Cas12 and PCR to detect *K. pneumonia*. It produces results in less than 2 h and requires less expensive equipment (Figure 1A). Furthermore, we tried to identify ESBLs by targeting *SHV* DNA fragments. Compared to the traditional detection methods, this trial may help community healthcare centers to accomplish nucleic acid detection.

## Results

### Establishment of PCR-LbCas12a detection targeting *Klebsiella pneumonia*

To find a suitable target for nucleic acid detection, we used blast to screen the most relevant gene as a target for nucleic acid detection. We downloaded 14,288 genomic sequences from NCBI and removed the sequences under 5.2 M, for this is a complete genomic DNA size for *K. pneumonia*. As a result, 2,024 sequences survived for further analysis. We found that *IF-2*, *16S RNA*, and *YP_005224572.1* compose 99.70% (2018/2024), 98.86% (2001/2024), and 99.70% (2018/2024) of all the sequences (Figure 1B). Therefore, they are suitable targets for nucleic acid detection. Considering the TTTV PAM sequences required for LbCas12a targeting, we designed five 36-nt long crRNAs which share 19 nt standards nucleotides and have 17 nt unique sequences to base-pair different *K. pneumoniae DNA* targets. K.P.-crRNA-1,2 target 16S RNA, K.P.-crRNA-3,4 target *YP_005224572.1*, and K.P.-crRNA-5 targets *IF-2*. We also screened the *K. pneumonia* genomes and found that these five crRNA targets separately 98.86% (2001/2024), 98.67% (1997/2024), 95.90% (1941/2024), 98.81% (2000/2024), and 99.90% (2022/2024) of the genomes (Figure 1C). To quickly test the detecting efficiency of the crRNAs, chemically synthetic single-stranded DNA targets were added to the LbCas12a system. Compared to the negative control, all crRNAs generated a significantly high level of FAM signals (Figure 1D). Further, the free FAM group generated by digesting probes can generate 488 nm fluorescent under 360 nM UV light exposure (Wang et al., 2022). We photographed the tube after the Cas12a reaction (Figure 1E). The K.P.-crRNA-2 and K.P.-crRNA-4 generate potent and stable FAM signals.

Next, we test the primers to amplify DNA fragments for crRNA-2 and crRNA-4. The amplification efficiency of *YP_005224572.1* primers is far more efficient than that of *16S* (Figure 1F). To test the specificity one step further, we blast the K.P.-crRNA-2,4, *16S*, *YP_005224572.1* in other *Klebsiella* strains (Supplementary Table 1). The results showed that the K.P.-crRNA-2 targets all of them, while the K.P.-crRNA-4 perfectly targets only *Klebsiella oxytoca. Klebsiella michiganensis*, *Klebsiella variicola*, and *Klebsiella Africana* have mutations on the targets, which might not be detected. As a result, K.P.-crRNA-4 and the primers targeting the *YP_005224572.1* gene are used to establish this system to detect the *K. pneumoniae* strain (Figure 1A).

### PCR-LbCas12a is sensitive and specific in *Klebsiella pneumonia* nucleic acid detection

**Figure 2:**
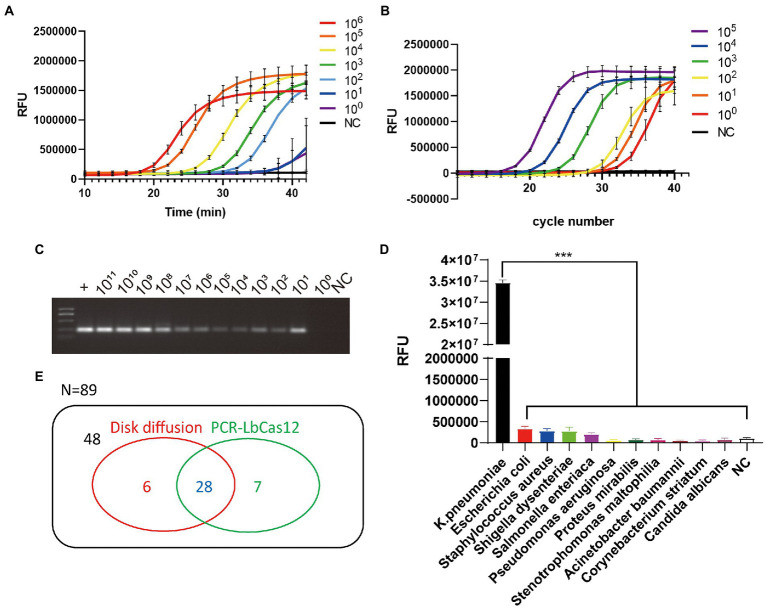
**(A)** The RFU signal generated by the PCR-LbCas12a system detecting the serially diluted standard *YP_005224572.1* DNA. **(B)** The qPCR results of serial diluted standard DNA samples. **(C)** 1% agarose gel electrophoresis of the PCR product from serial diluted standard DNA samples. **(D)** Different pathogens was detected by PCR-Cas12a system. Only *Klebsiella pneumoniae* was successfully detected. Data are mean ± s. d. of *n* = 3 biological independent experiments. **(E)** Venn diagram of results of testing 89 sputum samples in disk diffusion assay and PCR-LbCas12a workflow.

To explore the minimum amount of DNA sample required for nucleic acid detection, serial diluted standard DNA samples were to test PCR, qPCR, and PCR-LbCas12a techniques. Basic PCR exhibited positive signals when target DNA was as few as 10 copies (Figures 2A–C). In contrast, the LbCas12a system and qPCR can display a signal in 40 min when the copy number is as few as one single copy (Figure 2A). Next, to confirm if the detection system’s specificity targets only *K. pneumoniae*, we applied PCR-LbCas12a detection in 10 commonly seen pathogens in laboratory department, including *Escherichia coli* (*E. coli*), *Staphylococcus aureus* (*S. aureus*), *Shigella dysenteriae*, *Salmonella enterica*, *Pseudomonas aeruginosa*, *Proteus mirabilis*, *Stenotrophomonas maltophilia*, *Acinetobacter baumannii*, *Corynebacterium striatum*, and *Candida albicans* (fungi).

As a result, after 40 min PCR-Cas12 reactions, only *K. pneumonia* generated a significantly higher FAM signal (Figure 2D). The PCR-LbCas12a detection system is sensitive and specific.

### PCR-LbCas12a detects clinic samples

To apply this PCR-LbCas12a detection system to clinic use, we collected 89 sputum samples tested using disk diffusion. Total DNA was extracted from the samples. Then, PCR was processed and followed by LbCas12a incubation at 37°C for 20 min. Results were collected by photographing samples under UV light exposure (Supplementary Figure S1). Most of the positive and the negative samples were identical. Six samples were only positive in disk diffusion assay, and seven samples were only positive in PCR-LbCas12a workflow (Figure 2E). To verify the controversial samples, we PCR amplified them and processed Sanger sequencing. The Sanger sequence results showed that they are nearly 100% identical to the *K. pneumonia YP_005224572.1* gene fragment. Meanwhile, seven samples that are positive failed to be detected in this system. This failure might result from the corruption of the sputum samples, which were not well preserved after disk diffusion for diagnosis.

### Detection of *SHV* In ESBL-producing *Klebsiella pneumonia*

**Figure 3:**
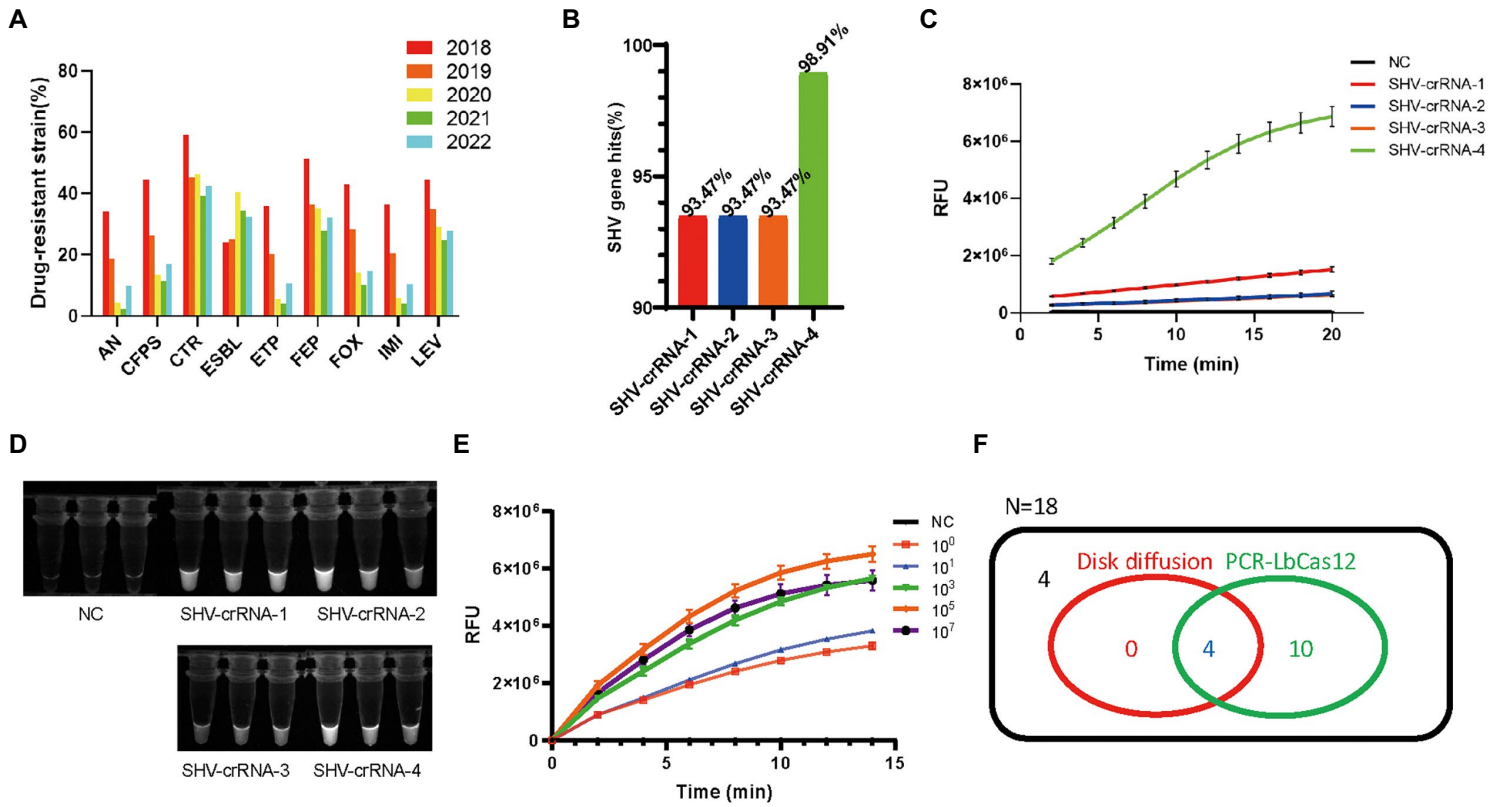
**(A)** The summary of drug-resistant *Klebsiella pneumonia* information in the Medical Laboratory Department of Shenzhen Luohu People’s Hospital from January 2018 to June 2022. **(B)** The percentage of *SHV* genes hit by four SHV-crRNAs. FAM signal, **(C)** and photograph under UV, **(D)** of four SHV-crRNAs activity in PCR-LbCas12a reaction. **(E)** The RFU signal generated by the PCR-LbCas12a system detecting the serially diluted standard *SHV* DNA. **(F)** Venn Diagram of results of testing ESBL of 18 K. pneumonia samples in PCR-LbCas12a and disk diffusion susceptibility test.

To start the task, we looked up the drug-resistant information of *K. pneumonia* in the medical laboratory department’s record since 2018. The ESBL-positive strains’ percentage is increasing from 22.9% (2018) to 40.3% (2020) and then remains at a relatively high level (Figure 3A). This information suggests that the identification of ESBL-positive strains is valuable. *SHV*-associated ESBLs are the most commonly reported groups, so we designed four crRNAs that target *SHV* to detect ESBLs. We also screened the 184 reported SHV genes by this four crRNAs, and found that SHV-crRNA1-4 target 93.47% (172/184), 93.47% (172/184), 93.47% (172/184), and 98.91% (182/184) of 184 SHV genes (Figure 3B).

Using the PCR-LbCas12a workflow, we tested the four crRNAs efficiencies. Although all the crRNAs are effective (Figures 3C, D), the SHV-crRNA-4 is the most efficient. The PCR-LbCas12a can test as few as one copy of the SHV-1 DNA fragment (Figure 3E).

Next, we detected the clinic samples. We collected 18 *K. pneumoniae* clinic samples that had been tested by disk diffusion susceptibility test. We tried it in our PCR-LbCas12a workflow and compared it with the clinic diagnosis (Figure 3F). All the ESBL (+) samples were positive in our workflow. Additionally, 10 more ESBL (−) were tested positive in PCR-LbCas12a detection. Sanger sequencing results suggest that three of them contain the *SHV-1* DNA fragment; the rest seven samples failed to be detected by PCR.

## Discussion

The excessive use of antibiotics increases drug-resistant bacteria. A recent discovery reveals that 73.1% of *K. pneumonia* are resistant to at least one antibiotic (Petrosillo et al., 2019; Sharahi et al., 2021). Multi-drug resistant and extensively drug-resistant *K. pneumonia* strains are increasing (Peng et al., 2020; Yan et al., 2021; Yang et al., 2021). Effectively controlling *K. pneumonia* requires the direct knowledge of drug-resistant information (Ludden et al., 2020).

The most used detection method is the disk diffusion antibiotic susceptibility test (Prastiyanto et al., 2020). However, the sensitivity and accuracy are only about 56% and 65% (Koyuncu and Haggblom, 2009). Furthermore, two rounds of tests are required to identify specific antibiotic information, which costs more time. In comparison, nucleic acid detection is more advantageous in terms of stability and accuracy. qPCR specifically and accurately identifies *K. pneumonia* from bacteria like *E. coli* and *S. aureus* (Kim et al., 2021) and its antibiotic-resistant gene (Castanheira et al., 2021). However, qPCR needs expensive equipment and skilled workers (Corman et al., 2020).

Recently, CRISPR-Cas12-mediated trans-collateral activity was widely applied to nucleic acid detection (Li et al., 2019; Ma et al., 2021; Selvam et al., 2021). The CRISPR-Cas12 detection system is accurate and specific and can also combine various readout and amplification technologies (Ali et al., 2020; Chen et al., 2020; Ramachandran et al., 2020).

In this study, we first established the PCR-LbCas12a system in this study to detect *K. pneumoniae* nucleic acid and *SHV* genes. We used PCR to harvest enough target DNA as a substrate for the LbCas12a reaction. The PCR-LbCas12a detection system can detect as low as only one copy of *K. pneumonia*. The results were highly consistent with the disk diffusion test. The clinic information on drug-resistant *K. pneumonia* showed that the ESBL (+) strains have been increasing over the past 5 years. We also tried the workflow to detect ESBL-resistant strains by detecting *SHV* fragments. The detection of *SHV* is successful. However, most of the positive strains are not ESBL (+) in disk diffusion tests. We speculate that this contradiction may result from the following reasons:

1. The disk diffusion might be too strict about detecting the *K. pneumonia* strains that are less resistant.
2. The *K. pneumonia* samples may not be well preserved to grow on the agar disk.
3. Many of the *SHV* variants do not encode ESBL enzymes.

Nevertheless, this workflow exhibits the potential to detect specific DNA fragments. Accurately detecting specific antibiotic-resistant strains needs more adjustment and knowledge of the mechanism. Compared to disk diffusion, PCR-LbCas12a detection, which takes no more than 2 h, is highly advantageous in time-consuming.

## Materials and methods

### Bioinformatics analysis and scripts

The source is downloaded from NCBI-genome. The R and Python scripts are prepared by Guoyu Peng. The detailed information is on https://github.com/GuoYu-Peng/GANAB_BLCA.

### Nucleic acid preparation

crRNAs were designed to target *16sRNA*, *YP_005224572.1*, and *IF-2* gene according to the protocol (Chen et al., 2018). RNA nucleotides were chemically synthesized without 5′-phosphorylation (Transheep, China). crRNA consists of 19 nt common sequences and 17 nt for recognizing target (Li et al., 2018). DNA and RNA sequences used in this manuscript are in the Supplementary material.

### DNA extraction and quantification

Clinical samples were swabs of sputa. According to the manufacturer’s protocol, swabs were dipped in cell lysate and processed genomic DNA extraction using the DNA extraction Kit (Tianlong science & technology, China). Extracted DNA samples were quantified by NanoDrop (Thermo Fisher Scientific, US) and preserved at −80°C before use.

### PCR and qPCR

PCR system was carried out in a 20 μl reaction system in the 0.2 ml EP tube. Each reaction contains 10 μl of PrimeSTAR (TAKARA, Japan) PCR premix, 1 μl of forward primer (10 nM) and 1 μl of reverse primer (10 nM), 10 ng of sample DNA, and ddH_2_O to supplement the volume to 20 μl. The PCR reactions were processed for 35 cycles on an Eppendorf thermocycler with denaturation at 94°C for 15 s, annealing at 58°C for 15 s, and extension at 72°C for 20 s. DNA electrophoresis was processed in 1% agarose gel in TAE buffer.

qPCR reactions were processed using Hieff UNICON Universal Blue qPCR SYBR Green Master Mix (Yeasen, China) on QuantStudio Dx (ABI, US). Program started with a 95°C for 2 min followed by 40 cycles of denaturation at 95°C for 10 s, annealing at 60°C for 10 s, and extension at 72°C for 15 s qPCR. Each reaction was repeated in three biologically independent experiments.

### PCR-LbCas12a detection

The LbCas12a detection was carried out in a 20 μl system. The system contains 2 μl Buffer 3 (NEB, US), 50 nM LbCas12a protein, 60 nM crRNA, and 30 nM labeled probe, and 100 ng purified PCR product. Samples were mixed and then incubated at 37°C, and signals were obtained from QuantStudio Dx (ABI, US) every minute for 20–60 min. Each reaction was repeated in three biologically independent experiments.

For clinic detection, samples were incubated at 37°C for 20 min, and then photographed under the UV light exposure. Two independent experiments were processed for each sample.

### Sample information

Sample information is available in Supplementary Table 2.

### Study approval

The Luohu Ethics Committee approved the project [LLBGS (2021) 020].

## Data availability statement

The datasets presented in this study can be found in online repositories. The names of the repository/repositories and accession number(s) can be found in the article/Supplementary material.

## Ethics statement

The Luohu Ethics Committee approved the project [LLBGS (2021) 020]. Written informed consent to participate in this study was provided by the participants’ legal guardian/next of kin.

## Author contributions

ShangW conceived this idea. ShangW, ShanW, and YT processed the experiments. GP analyzed the data. TH, XQ, and JW collected the clinic samples. XW extracted the DNA. YL, SZ and SoW offered the funding and the platform. All authors contributed to the article and approved the submitted version.

## Funding

This study was supported by the National Natural Science Foundation of China (81802741) to YL, Shenzhen Science and Technology Innovation Commission (RCJC20200714114557005) to SoW, The China Postdoctoral Science Foundation (2020 M682949) to ShangW, and The Guangdong Basic and Applied Basic Research Foundation (2020A1515110908) to SZ.

## Conflict of interest

The authors declare that the research was conducted in the absence of any commercial or financial relationships that could be construed as a potential conflict of interest.

## Publisher’s note

All claims expressed in this article are solely those of the authors and do not necessarily represent those of their affiliated organizations, or those of the publisher, the editors and the reviewers. Any product that may be evaluated in this article, or claim that may be made by its manufacturer, is not guaranteed or endorsed by the publisher.

## Footnotes

^1^ https://www.ncbi.nlm.nih.gov/pathogens/isolates#/refgene/SHV

## Abbreviations

PCR: Polymerase chain reaction
*K. pneumonia*: *Klebsiella pneumonia*
UTI: Urinary tract infection
BSI: Bloodstream infections
ESBL: Extended-spectrum β-lactamases
