# Supplementary material for: Detection of Klebsiella pneumonia DNA and ESBL positive strains by PCR-based CRISPR-LbCas12a system
Source: Front Microbiol. 2023 Feb 9;14:1128261. doi: 10.3389/fmicb.2023.1128261 (PMC9948084; doi:10.3389/fmicb.2023.1128261)
Supplement: Supplementary file 1 [file Data_Sheet_1.docx]

**Supplementary information**

**Materials and Methods**

Nucleic Acid Sequences:

The RNA sequences are as follows:

K.p.-crRNA-1：5’-AAU UUC UAC UGU UGU AGA UAC AUC CGA CUU GAC AGA-3’

K.p.-RNA-2：5’-AAU UUC UAC UGU UGU AGA UGA AUG CAG UUC CCA GGU-3’

K.p.-RNA-3：5’-AAU UUC UAC UGU UGU AGA UUC CAC ACU UCC GGA UAG-3’

K.p.-RNA-4：5’-AAU UUC UAC UGU UGU AGA UCC GCU GGU ACC GGC AUC-3’

K.p.-RNA-5：5’-AAU UUC UAC UGU UGU AGA UCG UGC UGG AUA GCU UCG-3’

ESBL-crRNA-1: 5’-AAU UUC UAC UGU UGU AGA UUG CGC CAG AUC GGC GAC-3’

ESBL-crRNA-2: 5’-AAU UUC UAC UGU UGU AGA UGC GCC AGA UCG GCG ACA-3’

ESBL-crRNA-3: 5’-AAU UUC UAC UGU UGU AGA UCG CCA GAU CGG CGA CAA-3’

ESBL-crRNA-4: 5’-AAU UUC UAC UGU UGU AGA UCC AGC GGU CAA GGC GGG-3’

Probe sequence and modification is 5’-FAM-TTTTTT-BHQ-3’.

DNA targets for crRNA testing were synthesized by Sangon Corp (China). The sequences are as follows:

K.p.-Target-1: 5’-GGT CTG TCA AGT CGG ATG TGA AAT C-3’

K.p.Target-2: 5’-TCA ACC TGG GAA CTG CAT TCG AAA CTG-3’

K.p.Target-3: 5’-GGG CTA TCC GGA AGT GTG GAT AAA CG-3’

K.p.Target-4: 5’-GCA GAT GCC GGT ACC AGC GGG AAA AC-3’

K.p.Target-5: 5’-TAT CGA AGC TAT CCA GCA CGC TAA AGC-3’

Primer used for DNA amplification:

qKP-F: 5’- TGG ACA AAG ACT GAC GCT CA -3’,

qKP-R: 5’- TGC TCG GTG TTA TTG AGA-3’.

qKP-F: 5’-TCT CAA TAA CAC CGA GCA-3

qKP-R: 5’-TGC TCG GTG TTA TTG AGA-3’.

SHV-qF1: ATAACAGCGCCGCCAATCT

SHV-qR1: GAAGCGCCTCATTCAGTTCC

SHV-qF2: CATGAGCGATAACAGCGCC

SHV-qR2: AGCGCCTCATTCAGTTCCG

Standard DNA carrying *K. pneumoniae* 16sRNA, YP_005224572.1 and IF-2 gene fragments were synthesized and cloned to pUC-19 vector. Full sequences are as follows:

K.P.-16SRNA: 5’-AGGAAGGCCGGTGAGGTTAATAACCTCTCGATTGACGTTACCCGCAGAAGAAGCACCGGCTAACTCCGTGCCAGCAGCCGCGGTAATACGGAGGGTGCAAGCGTTAATCGGAATTACTGGGCGTAAAGCGCACGCAGGCGGTCTGTCAAGTCGGATGTGAAATCCCCGGGCTCAACCTGGGAACTGCATTCGAAACTGGCAGGCTAGAGTCTTGTAGAGGGGGGTAGAATTCCAGGTGTAGCGGTGAAATGCGTAGAGATCTGGAGGAATACCGGTGGCGAAGGCGGCCCCCTGGACAAAGACTGACGCTCAGGTGCGAAA -3’

K.P.-YP_005224572.1: 5’-CGAGGTTTACGTCTCAACCGGCTGGGGATCCACCACGAGCGGCTGCCGCCCGGGCGGCGCACCTCTTATCCACACGCGGAGAGCGATGAGGAAGAGTTCATCTACGTGCTGGAGGGCTATCCGGAAGTGTGGATAAACGGCTATCTCTGGAAGCTGGAGCCCGGCGACAGCGTGGGTTTTCCCGCTGGTACCGGCATCTGCCACACCTTTCTCAATAACACCGAGCAGGAAGTTCGTCTG-3’

K.P.-IF-2:

5’-CTGGTGGTGGGCGAGGCCAACAAGAAGTACAACCGCATTTATTACCGGCATGATCACCTTCCTGGATACCCCGGGCCACGCCGCGTTTACCTCCATGCGTGCTCGTGGCGCGCAGGCGACGGATATCGTGGTTCTGGTGGTGGCGGCAGACGACGGCGTGATGCCGCAGACTATCGAAGCTATCCAGCACGCTAAAGCGGCGCAGGTACCGGTGGTAGTGGCGGTGAACAAGATCGATAAGCCAGAAGCCGATCCGGATCGCGTGAAGAACGAACTGTCCCAGTACGGCATCCTGCCGGAAGAGTGGGGCGGCGAGAGCCAGTTCGTCCACGTTTCCGCGAAAGCGGGTACCGGCATCGACGAC (IF-2 GenBank: CP052761.1; LOCUS: CP052761; gene ID: inf B 1,329-1,647)-3’

ESBL-SHV:

5’-GCCATTACCATGAGCGATAACAGCGCCGCCAATCTGCTGCTGGCCACCGTCGGCGGCCCCGCAGGATTGACTGCCTTTTTGCGCCAGATCGGCGACAACGTCACCCGCCTTGACCGCTGGGAAACGGAACTGAATGAGGCGCTTCCCGGCGACGCCCGCGACACCACTACCCCGGCCAGCATGGCCGCGACCCTGCGCAA-3’

Python or R Script are as follows:

1. **K.P. genomes were filtered by kp_genome_crRNA_stats.R**

library(here)

library(tidyverse)

library(ggthemes)

fasta_dir <- here("kp_fasta")

match_path <- file.path(fasta_dir, "kp_genome_crRNA.csv")

match_data <- read_csv(match_path) %>%

filter(seq2_len >= 5000000)

write_csv(match_data, file.path(fasta_dir, "kp_genome_crRNA_pass.csv"))

plot_data1 <- count(match_data, seq1, hit)

plot1 <- ggplot(plot_data1) +

geom_bar(aes(x = seq1, y = n, fill = hit), stat = "identity", position = "fill") +

labs(x = "Seq", y = "Fraction", fill = "Hit") +

theme_classic()

ggsave("kp_genome_match_stats.pdf", plot = plot1, device = "pdf", path = fasta_dir)

1. **The percentage of hitting K.P.-crRNAs were given by kp_genome_crRNA_mach.py**

import pathlib as pl

from Bio import SeqIO

from Bio.Seq import Seq

def tr_back(seq: str):

x = Seq(seq)

return str(x.back_transcribe().reverse_complement())

def find_hit(seq1: Seq, seq2):

if seq2.find("U") != -1:

seq2 = tr_back(seq2)

seq1_rv = seq1.reverse_complement()

if (seq1.find(seq2) != -1) or (seq1_rv.find(seq2) != -1):

return "yes"

else:

return "no"

fasta_dir = pl.Path("../kp_fasta")

fasta_path1 = pl.Path(fasta_dir, "kp_genomes.fasta")

hit_path = pl.Path(fasta_dir, "kp_genome_crRNA.csv")

crRNAs = {"crRNA-1": "ACAUCCGACUUGACAGA", "RNA-2": "GAAUGCAGUUCCCAGGU", "RNA-3": "UCCACACUUCCGGAUAG",

"RNA-4": "CCGCUGGUACCGGCAUC", "RNA-5": "CGUGCUGGAUAGCUUCG"}

fa_open = open(fasta_path1, "r")

fa_list = list(SeqIO.parse(fa_open, "fasta"))

fa_open.close()

with open(hit_path, "w") as fh:

fh.write("seq1,seq2,seq2_len,hit\n")

for each_id, each_seq in crRNAs.items():

for i, each_rc in enumerate(fa_list):

seq1 = each_rc.seq

id1 = each_rc.id

len1 = len(seq1)

hit_result = find_hit(seq1, each_seq)

fh.write(f"{each_id},{id1},{len1},{hit_result}\n")

Figure S1


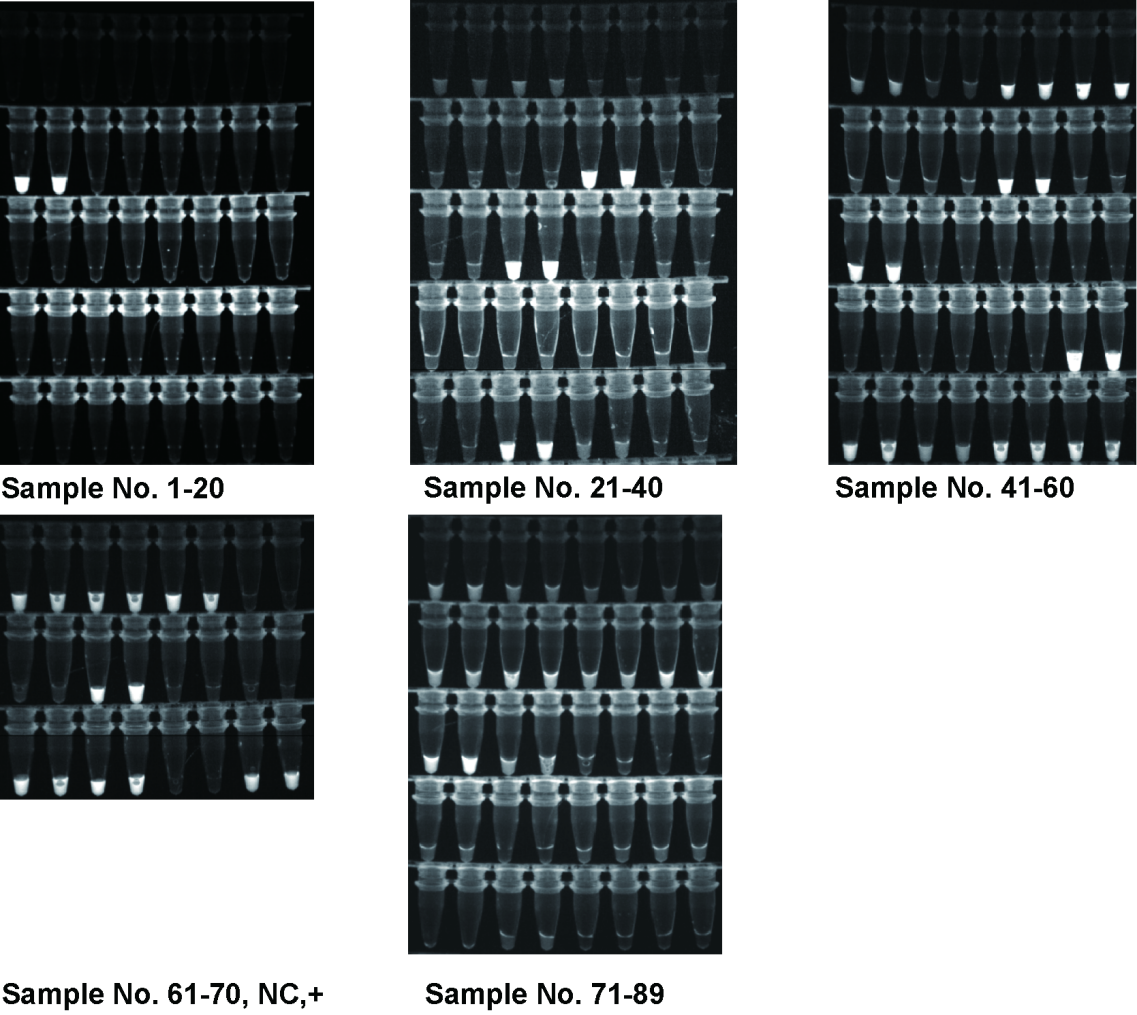


**Figure S1**. The results of clinic samples in PCR-LbCas12a workflow.

|  | 16S | YP_005224572.1 | K.P-crRNA-2 | K.P-crRNA-4 |
| --- | --- | --- | --- | --- |
| *Klebsiella pneumoniae* | + | + | + | + |
| *Klebsiella oxytoca* | + | + | + | - |
| *Klebsiella aerogenes* | + | + | + | + |
| *Klebsiella michiganensis* | + | + | + | +/- |
| *Klebsiella variicola* | + | + | + | +/- |
| *Klebsiella quasipneumoniae* | + | - | + | - |
| *Klebsiella grimontii* | + | - | + | - |
| *Klebsiella variicola* CAG:634 | + | - | + | - |
| *Klebsiella africana* | + | + | + | +/- |
| *Klebsiella huaxiensis* | + | - | + | - |
| *Klebsiella quasivariicola* | + | + | + | - |

**Table1**. The blast results of *16S*, *YP_005224572.1* and K.P.-crRNA-2,4 in different *Klebsiella*.

| **No.** | **Gender** | **Age** | ***K.Pneumonia*** | **No.** | **Gender** | **Age** | ***K.Pneumonia*** | **No.** | **Gender** | **Age** | ***K.Pneumonia*** |
| --- | --- | --- | --- | --- | --- | --- | --- | --- | --- | --- | --- |
| 1 | F | 2 | - | 31 | F | 76 | - | 61 | M | 75 | + |
| 2 | M | 59 | - | 32 | M | 58 | - | 62 | M | 55 | + |
| 3 | M | 89 | - | 33 | M | 60 | - | 63 | M | 55 | + |
| 4 | F | 85 | - | 34 | F | 88 | - | 64 | F | 93 | + |
| 5 | M | 50 | - | 35 | M | 47 | - | 65 | F | 79 | + |
| 6 | F | 68 | - | 36 | M | 76 | - | 66 | F | 5 | + |
| 7 | M | 87 | - | 37 | M | 86 | - | 67 | F | 53 | + |
| 8 | M | 11d | - | 38 | M | 75 | + | 68 | M | 91 | + |
| 9 | F | 3 | - | 39 | F | 58 | - | 69 | F | 47 | + |
| 10 | M | 82 | - | 40 | M | 72 | - | 70 | F | 89 | + |
| 11 | M | 9 | - | 41 | M | 64 | - | 71 | M | 87 | + |
| 12 | M | 60 | - | 42 | M | 72 | - | 72 | F | 82 | + |
| 13 | M | 70 | - | 43 | M | 70 | - | 73 | M | 55 | + |
| 14 | F | 45 | - | 44 | M | 52 | + | 74 | M | 60 | + |
| 15 | F | 87 | - | 45 | M | 42 | - | 75 | M | 74 | + |
| 16 | F | 89 | - | 46 | F | 47 | - | 76 | M | 82 | + |
| 17 | M | 80 | - | 47 | M | 42 | + | 77 | F | 75 | + |
| 18 | M | 83 | - | 48 | F | 69 | + | 78 | M | 70 | + |
| 19 | M | 89 | - | 49 | F | 43 | - | 79 | M | 51 | + |
| 20 | F | 49 | - | 50 | M | 47 | - | 80 | M | 82 | + |
| 21 | M | 42 | - | 51 | M | 65 | - | 81 | F | 70 | + |
| 22 | F | 58 | - | 52 | F | 1month | - | 82 | M | 40 | - |
| 23 | M | 72 | - | 53 | M | 25 | - | 83 | M | 92 | + |
| 24 | F | 64 | - | 54 | M | 9 | - | 84 | F | 85 | - |
| 25 | M | 50 | - | 55 | F | 57 | - | 85 | M | 48 | + |
| 26 | F | 43 | - | 56 | M | 49 | + | 86 | M | 67 | + |
| 27 | M | 88 | + | 57 | M | 55 | + | 87 | M | 27 | + |
| 28 | F | 21h | - | 58 | M | 105 | + | 88 | M | 89 | - |
| 29 | M | 82 | - | 59 | F | 69 | + | 89 | M | 86 | - |
| 30 | F | 74 | - | 60 | M | 77 | + |  |  |  |  |

**Table 2.** Sputum samples are obtained from each donor and stored at 4°C until processing. Nucleic acid extracts derived from 89 sputum samples are from Medical Laboratory of Shenzhen Luohu People's Hospital. The basic information is available (mean age, 63.7 ± 22.3 years, remove the data of three babies under one years; gender, 57 men and 32 women; ). All samples are collected from June to July 2022.
